# Aerobic Growth of *Rhodococcus aetherivorans* BCP1 Using Selected Naphthenic Acids as the Sole Carbon and Energy Sources

**DOI:** 10.3389/fmicb.2018.00672

**Published:** 2018-04-12

**Authors:** Alessandro Presentato, Martina Cappelletti, Anna Sansone, Carla Ferreri, Elena Piacenza, Marc A. Demeter, Silvia Crognale, Maurizio Petruccioli, Giorgio Milazzo, Stefano Fedi, Alexander Steinbüchel, Raymond J. Turner, Davide Zannoni

**Affiliations:** ^1^Department of Pharmacy and Biotechnology, University of Bologna, Bologna, Italy; ^2^Department of Biological Sciences, University of Calgary, Calgary, AB, Canada; ^3^ISOF, Consiglio Nazionale delle Ricerche, Bologna, Italy; ^4^Department for Innovation in Biological, Agro-food and Forest Systems (DIBAF), University of Tuscia, Viterbo, Italy; ^5^Institute of Molecular Microbiology and Biotechnology, University of Münster, Münster, Germany; ^6^Department of Environmental Sciences, King Abdulaziz University, Jeddah, Saudi Arabia

**Keywords:** *Rhodococcus aetherivorans*, naphthenic acids, stress response, β-oxidation, transmission electron microscopy, fatty acids methyl esters, inclusion bodies

## Abstract

Naphthenic acids (NAs) are an important group of toxic organic compounds naturally occurring in hydrocarbon deposits. This work shows that *Rhodococcus aetherivorans* BCP1 cells not only utilize a mixture of eight different NAs (8XNAs) for growth but they are also capable of marked degradation of two model NAs, cyclohexanecarboxylic acid (CHCA) and cyclopentanecarboxylic acid (CPCA) when supplied at concentrations from 50 to 500 mgL^-1^. The growth curves of BCP1 on 8XNAs, CHCA, and CPCA showed an initial lag phase not present in growth on glucose, which presumably was related to the toxic effects of NAs on the cell membrane permeability. BCP1 cell adaptation responses that allowed survival on NAs included changes in cell morphology, production of intracellular bodies and changes in fatty acid composition. Transmission electron microscopy (TEM) analysis of BCP1 cells grown on CHCA or CPCA showed a slight reduction in the cell size, the production of EPS-like material and intracellular electron-transparent and electron-dense inclusion bodies. The electron-transparent inclusions increased in the amount and size in NA-grown BCP1 cells under nitrogen limiting conditions and contained storage lipids as suggested by cell staining with the lipophilic Nile Blue A dye. Lipidomic analyses revealed significant changes with increases of methyl-branched (MBFA) and polyunsaturated fatty acids (PUFA) examining the fatty acid composition of NAs-growing BCP1 cells. PUFA biosynthesis is not usual in bacteria and, together with MBFA, can influence structural and functional processes with resulting effects on cell vitality. Finally, through the use of RT (Reverse Transcription)-qPCR, a gene cluster (*chcpca*) was found to be transcriptionally induced during the growth on CHCA and CPCA. Based on the expression and bioinformatics results, the predicted products of the *chcpca* gene cluster are proposed to be involved in aerobic NA degradation in *R. aetherivorans* BCP1. This study provides first insights into the genetic and metabolic mechanisms allowing a *Rhodococcus* strain to aerobically degrade NAs.

## Introduction

Naphthenic acids (NAs) (C_n_H_2n_+_z_O_2_) are defined as a complex mixtures of organic acids mainly comprised of saturated aliphatic and alicyclic carboxylic acids (Whitby, 2010). They are naturally occurring in non-conventional hydrocarbon deposits, including bituminous oil sands, and are major contributors to the toxicity of oil sands-process affected waters (OSPW) – the tailings wastes produced during bitumen extraction. Because of the acute and chronic toxicity exerted by NAs to a wide range of aquatic organisms, birds and mammals, there are concerns about the environmental impacts of the release of OSPW (Allen, 2008; Whitby, 2010). The toxicity effects of NAs are a result of their surfactant properties. Acting through a non-specific mode of action called narcosis, NAs can disrupt the lipid bilayer of membranes and change membrane properties (Frank et al., 2008). The degree of toxicity is directly related to NA molecular structure. Lower molecular weight acids (particularly acyclic compounds) often demonstrate higher toxicity as they can more easily penetrate and disrupt cell membranes as compared to higher molecular weight compounds with increasing instances of alkyl side chain branches and ring structures (Jones et al., 2011). Nevertheless, oil sands tailings ponds (discarded bodies of OSPW, sand, clay and residual bitumen) were shown to contain microbial communities able to biodegrade NAs (Lai et al., 1996). For this reason, several studies on NA biodegradation using indigenous OSPW bacteria applied in batch-reactors or engineered biofilm systems were performed in the last two decades. Several abiotic factors have proven to affect the NA biodegradation efficiency of OSPW derived microbial communities including temperature, oxygen, phosphate and nitrogen concentrations, contaminant structure (and by extent toxicity) along with the presence of additional nutrients (Lai et al., 1996; Clemente et al., 2004; Smith et al., 2008; Johnson et al., 2010; Demeter et al., 2014, 2015).

As pure bacterial cultures, members of the genera *Corynebacterium, Arthrobacter, Acinetobacter, Alcaligenes, Alkanivorax*, and *Pseudomonas* were able to utilize cyclohexanecarboxylic acid (CHCA) as carbon sources for growth (Blakley, 1974, 1978; Rho and Evans, 1975; Blakley and Papish, 1982; Whitby, 2010). In these studies, the detection of specific metabolic intermediates suggested possible metabolic pathways for CHCA utilization, namely: (1) via β-oxidation, (2) via a pathway similar to benzoate degradation, and (3) via the aromatization of the cyclohexane ring. The β-oxidation pathway was also found to drive the degradation of aromatic alkanoic NA butylphenylbutanoic acid (BPBA) in *Pseudomonas putida* KT2440 and *Mycobacterium* sp. strain IS2.3 (Johnson et al., 2011, 2012); while in *Rhodopseudomonas palustris*, CHCA was found to be degraded through metabolic reactions involved in anaerobic metabolism of benzoate, implying the involvement of *bad*/*ali* gene products (Pelletier and Harwood, 2000). In *Cupriavidus gilardii*, homologs to *bad/ali* genes were predicted via *in silico* analysis to be involved in the peripheral ring-cleavage pathway for NA degradation (Wang et al., 2015). In *Arthrobacter* sp. strain ATCC51369, previously named as *Corynebacterium cyclohexanicum*, a *pobA* gene coding for a 4-hydroxybenzoate hydroxylase was reported to be involved in CHCA metabolism along with the flanking genes encoding a *pobA* gene regulator (Iwaki et al., 2005). These data, taken together, make us to conclude that our present knowledge of the biodegradation gene clusters involved in NA degradation is quite limited.

Recently, members of the genus *Rhodococcus* were isolated from OSPW microbial communities and some preliminary indications were given on their capacity to degrade NAs (Koma et al., 2004; Golby et al., 2012; Demeter et al., 2015). *Rhodococcus* spp. are known for their broad catabolic diversity and their extraordinary tolerance to various environmental stress such as high doses of toxic compounds, desiccation, carbon starvation, UV irradiation and osmotic stress (Cappelletti et al., 2016). *Rhodococcus* spp. exemplary tolerance to organic contaminants has been linked to strategic modification of fatty acid composition (de Carvalho et al., 2005). Several studies have also reported the ability of *Rhodococcus* spp. to simultaneously degrade recalcitrant organics while accumulating polymeric lipids such as polyhydroxyalkanoates (PHA) and triacylglycerols (TAGs) within the cells (Alvarez et al., 1997; Hernández et al., 2008). These storage lipophilic compounds have multiple applications in the production of food additives, lubricants, oleochemicals, biofuels, and bioplastics (Alvarez et al., 2013; Bugnicourt et al., 2014).

Here, we report for the first time on the capacity of *R. aetherivorans* BCP1 (hereinafter named as BCP1) to grow utilizing representative aliphatic and alicyclic NAs. We further analyzed in detail the use of two such model NAs, cyclohexanecarboxylic acid (CHCA) and cyclopentanecarboxylic acid (CPCA) as carbon and energy sources for BCP1 aerobic growth. We conclude that although CHCA and CPCA result to be toxic to BCP1 cells when used at high concentrations (>200 mg L^-1^), degradation of these two NAs took place in parallel with (i) a change in the morphology and the accumulation of electron-dense inclusion bodies, (ii) a change in cellular fatty acid composition, and (iii) the transcriptional induction of a gene cluster (*chcpca*) encoding enzymes involved in the β-oxidation reaction pathway. This study on *R. aetherivorans* BCP1 cell growth and response to the toxicity exerted by NAs, provides the first set of genetic data on bacterial NAs degradation and highlights the possibility to bioconvert toxic NAs into valuable products such as polymeric lipids.

## Materials and Methods

### Bacterial Cultures and Growth Conditions

*Rhodococcus aetherivorans* BCP1 (DSM 44980) was initially isolated from a butane-utilizing microbial consortium able to co-metabolically degrade chloroform in batch slurry reactors (Frascari et al., 2005). For the growth and degradation assays, BCP1 strain was firstly pre-cultured in 250 mL Erlenmeyer Baffled Flasks for 48 h, containing 25 mL of LB medium [composed of (g L^-1^) NaCl, 10; Yeast Extract, 5; Tryptone, 10]. After this, the cells were inoculated in 50 mL of Mineral Salts Medium (MSM) (Schlegel et al., 1961) supplied with the SL6 source of trace elements (Pfennig, 1974), at an initial OD_600_ of 0.05. The following carbon and energy sources were added to MSM: 8XNA mixture at a working concentration of 500 mg L^-1^ (Supplementary Table S1, Demeter et al., 2015), glucose and succinate at 0.1% w/v, which are standard concentrations previously shown to allow BCP1 cultures to reach OD_600_ values of 0.5–0.7 (Cappelletti et al., 2011, 2015). Select incubations received CHCA or CPCA supplied at concentrations of 50, 100, 200, and 500 mg L^-1^, in lieu of the 8XNA mixture. Individual NA stock solutions were prepared at a concentration of 10 mg mL^-1^ in 0.1 M NaOH solution. Solution pH was adjusted to around 10, to dissolve the NAs as sodium naphthenates, which are soluble in water (Clemente and Fedorak, 2005; Quesnel et al., 2011; Demeter et al., 2015). The sodium naphthenates solutions were filter sterilized through a 0.20 μm pore sized filter before addition to the MSM medium. Each culture was incubated aerobically at 30°C on a horizontal rotary shaker (150 rpm). To evaluate the bacterial growth rate, the change in optical density at 600 nm (OD_600_) was measured every 3 (CHCA or CPCA cultures) or 24 (8XNAs cultures) hours. For CHCA and CPCA, final cell dry weights (mg cdw) were also measured after 44 h of growth by drying the grown biomass on filters at 70°C for 2 h. Data is reported as the mean of three biological replicates with standard deviation. All the reagents were purchased from Sigma-Aldrich.

### Reverse Transcription (RT-) PCR and Quantitative Real Time PCR (qRT-PCR) Analysis

Total RNA was isolated from BCP1 cells grown (up to exponential phase) in 100 mL of MSM supplied with CHCA (500 mg L^-1^), CPCA (500 mg L^-1^), glucose (0.1% w/v) or succinate (0.1% w/v) as sole carbon and energy source. The procedures that were used for total RNA extraction, RNA treatment with RNase-free DNase, and cDNA synthesis are described by Cappelletti et al. (2015). In order to study the transcriptional induction of the genes potentially coding for enzymes involved in NA degradative pathways, one tenth of the cDNA mixture was amplified using the sets of primers listed in Supplementary Table S3. After an initial incubation at 94°C for 2 min, 25 cycles of the following temperature program were used: 94°C for 30 s, 60°C for 30 s, and 72°C for 30 s. Negative controls were performed by omitting the reverse transcriptase in RT-PCR experiments using the same temperature program and the same primer sets for 30 cycles of amplification.

In order to assess the transcriptional level of the eight genes included in the *chcpca* cluster, (comparing CHCA and CPCA incubations to succinate and glucose controls), Reverse Transcriptase- quantitative PCR (RT-qPCR) analyses were performed as previously reported (Cappelletti et al., 2015) using the sets of primers listed in Supplementary Table S2. The ΔΔ*C*t method with 16S rRNA as a reference gene was used to determine relative abundance of target transcripts. Relative gene expression was reported as the change (*n*-fold) determined from the mean normalized expression relative to the mean normalized expression of the reference gene (ΔΔ*C*t method). Data are expressed as mean ± standard deviation derived from at least three independent experiments. For each RNA preparation, at least three independent real-time PCR experiments were conducted.

### Analysis of NA Removal

For each time point considered in this study, 1 mL of culture was collected and centrifuged (3 min at 15,700 × *g*) to collect BCP1 biomass. The recovered spent medium was then processed as described by Demeter et al. (2015) to extract NAs present in solution. Briefly, the internal standard 4-phenyl butyric acid (4-PBA) was added to spent medium at a concentration of 1.3 g L^-1^ (Sigma-Aldrich) and the resulting sample was acidified to pH 2 with HCl (5.2 M), and transferred into glass vials sealed with Teflon-lined lids. NAs were then extracted into two volumes of dichloromethane by mixing the sample at room temperature for 3 h. The organic phase, now containing NAs, was recovered using phase separator filter papers (GE Whatman) and condensed with a rotary evaporator. NAs were then derivatized as trimethylsilylates at 60°C for 10 min using *N,O*-bis(trimethylsilyl)trifluoroacetamide (Fluka; Quesnel et al., 2011). The relative abundance of each individual NA was determined via gas chromatography coupled to a flame ionization detector (GC-FID, Agilent 7890). The GC-FID was equipped with an Agilent HP-5 column (30 m), configured as follows: 4 μL injection volume, 2:1 injector split ratio. The oven temperature program consisted of a 2 min hold at 70°C, followed by an increase to 230°C at a ramp rate of 5°C/min, which was held for a final 2 min. In order to evaluate the potential for abiotic loss of NAs, as well as to normalize their relative abundance over the incubation time, control samples were evaluated using growth medium containing autoclaved BCP1 biomass and the 8XNA mixture. The data has been reported as the mean of at least three biological replicates with standard deviation. Note that observed removal of NAs recorded in this assay is a loss of the parent compound alone – no indications of further degradation are obtained.

### Staining Procedure and Confocal Laser Scanning Microscopy (CLSM)

Heat-fixed BCP1 cells grown (exponential phase) in MSM under nitrogen limiting conditions (0.4 g L^-1^ NH_4_Cl) in the presence of glucose (0.1% v/v), CHCA or CPCA (500 mg L^-1^) were collected and stained with a 1% aqueous solution of the lipophilic fluorescent dye, Nile Blue A (Sigma-Aldrich) at 55°C for 10 min, as described by Legat et al. (2010). Nile Blue A staining was previously described to allow the visualization of intracellular lipid inclusions through fluorescence microscopy (Oshiki et al., 2011; Weissgram et al., 2015). Excess Nile Blue A stain was removed by washing the stained cells with both distilled water and an 8% acetone solution for 1 min (Legat et al., 2010). The smear was air-dried and covered with a glass cover slip prior to imaging, which was performed by using a Leica DM IRE2 fluorescence microscope with a 20× objective. Further image processing was conducted using Imaris x64 Software (Bitplane Scientific Software, South Windsor, CT, United States).

### Transmission Electron Microscopy (TEM) Observations

The BCP1 strain was cultured in MSM under standard (1 g L^-1^) or limiting (0.4 g L^-1^) conditions of nitrogen (NH_4_Cl), which was amended with glucose (0.1% v/v), CHCA or CPCA (500 mg L^-1^) as carbon and energy sources. Exponential growth phase BCP1 cells were collected and washed twice with phosphate-buffered saline [PBS; containing (g L^-1^) NaCl, 8; KCl, 0.2; Na_2_HPO_4_, 1.44; KH_2_PO_4_, 0.24] pH 7.4. Post-washing the cells were first fixed for 2 h at room temperature with a solution containing glutaraldehyde (2.5%), HEPES (50 mM) and MgCl_2_ (1 mM) pH7 and subsequently rinsed three times with sodium cacodylate buffer (0.1 M) pH 7. An additional fixation step was conducted with a solution containing osmium tetroxide (2%) and sodium cacodylate (0.1 M) for 1 h at room temperature. Finally, increasing concentrations of water/ethanol solutions (30, 50, 70, 85, 95, and 100%) were added to the cells to dehydrate them. In particular, each water/ethanol solution was used to wash the cells for 30 min. After this, each cell pellet was washed for three times (10 min each) with 1 mL of propylene oxide (>99.5% v/v). Bacterial cells were then infiltrated over night at room temperature with a mixture of propylene oxide:epoxy resin (Fluka) 1:1, which was prepared as described by the manufacturer’s protocol. Thereafter, BCP1 cells were further infiltrated by epoxy resin (100%) for 4.5 h at room temperature. Resin polymerization occurred during further incubations at 45 and 60°C for 12 and 24 h, respectively. The resulting resin blocks were ultra-thin sectioned, and mounted onto copper slot grids (Electron Microscopy Sciences). Thin sections were imaged using a Hitachi H7650 TEM operating at 80 kV. All the reagents were purchased from Sigma-Aldrich. Cell size measurements were conducted on a total of 150 cells from 10 different ocular fields (pictures).

### Flow Cytometry

Flow cytometry experiments were done according to Ziglio et al. (2002). Briefly, cell pellets were diluted with Tricine buffer (0.1 M Tricine, 10 mM MgCl_2_ at pH 7.4 and filtered using a pore size of 0.22 μm) and stained with SYBR Green I (SYBR I) and propidium iodide (Molecular Probes Inc., Eugene, OR, United States) by adding 10 μl of both fluorochromes per ml of sample. The cytofluorimetry count of viable and dead cells is based on their membrane integrity as determined by staining with SYBR I and propidium iodide, two high-affinity nucleic acid dyes that give bright staining of cells. Although no specific information is available on the structure and chemical properties of SYBR I or on its mode of binding to DNA, it has widely been shown to be capable of staining all cells, living or dead (Nebe-von-Caron et al., 2000). Conversely, the polarity of propidium iodide allows it to penetrate only leaky cell membranes, which are characteristic of dead or damaged cells. In dead cells, the simultaneous presence of SYBR I and propidium iodide activates energy transfer between SYBR I and propidium iodide so that the fluorescence emission of SYBR I is no longer visible. In this way it is possible to distinguish viable cells (green fluorescent) from dead cells (red fluorescent). Samples were analyzed with a Bryte-HS flow cytometer (Bio-Rad, Hercules, CA, United States) with a high light-scattering sensitivity and equipped with a short-arc xenon lamp (Hamamatsu). Samples were excited at 470–490 nm, and fluorescence emission was detected at 515–565 nm for SYBR I (λ = 488 nm, λ = 525 nm) and at 590–720 nm for propidium iodide (λ = 530 nm, λ = 620 nm). Samples were analyzed for 3–4 min at a flow rate of 1.5 μl min^-1^ for cell enumeration. Data acquisition was triggered to reduce interference by non-fluorescent particles (debris), while photomultiplier gains were set in the logarithmic mode, and data were recorded as list mode files by WinBryte software (Bio-Rad).

### Lipid Extraction and Fatty Acid Analysis of *R. aetherivorans* BCP1

The pellets of bacteria grown on glucose 0.1% w/v, CHCA 500 mg L^-1^ or CPCA 500 mg L^-1^ until exponential phase (ca. 100 mg, wet weight) were added to tridistilled H_2_0 (4 mL) and extracted with 2:1 chloroform/methanol (5 × 6 mL) according to the Folch method in order to isolate lipids (Folch et al., 1957). The organic layer was collected and dried over anhydrous Na_2_SO_4_ then evaporated under vacuum to dryness and weighted. Analytical thin-layer chromatography (TLC) was performed on Merck silica gel 60 plates (0.25 mm thickness) as previously described (Sansone et al., 2013) and spots were detected by spraying the plate with cerium ammonium sulfate/ammonium molybdate reagent and revealed by heating the plate. TLC monitoring (*n*-hexane/Et_2_0 8:2) revealed that the extracts of BCP1 cells cultivated in presence of CHCA and CPCA, were composed by phospholipids, triglycerides and cholesterol, whereas cells grown on glucose mainly presented phospholipids, and traces of triglycerides and cholesterol. The lipid extracts were treated with 1 mL of 0.5 M solution of KOH in methanol for the transesterification, in order to perform the conversion of fatty acid-containing esters (acylglycerols, phospholipids) into the corresponding methyl esters for GC analysis. The reaction was stirred for 30 min at room temperature and quenched by adding brine (1 mL). FAMEs were extracted with *n*-hexane (3 × 2 mL), the organic phases were collected, dried over anhydrous Na_2_SO_4_ and evaporated to dryness. The residue of each sample was dissolved in 20 μL of *n*–hexane and 1 μL was injected in Gas Chromatography (GC) with a split ratio of 50:1 for Fatty Acid Methyl Esters (FAME) analysis. The GC (Agilent 6850, Milan) was equipped with a 60m × 0.25mm × 0.25 μm (50%-cyanopropyl)-methylpolysiloxane column (DB23, Agilent, United States), and a flame ionization detector with the following oven program: temperature started from 165°C, held for 3 min, followed by an increase of 1°C/min up to 195°C, held for 40 min, followed by a second increase of 10°C/min up to 240°C, and held for 10 min. A constant pressure mode (29 psi) was chosen with helium as carrier gas. The FAME were also analyzed by GC-MS (Thermo Scientific Trace 1300) equipped with a 15m × 0.25 mm × 0.25 μm TG-SQC 5% phenyl methyl polysiloxane column, with helium as carrier gas, coupled to a mass selective detector (Thermo Scientific ISQ) with the following oven program: temperature started at 80°C, maintained for 2 min, increased at a rate of 15°C/min up to 140°C, increased at a rate of 5°C/min up to 280°C and held for 10 min (Ferreri et al., 2017).

Statistical analysis was performed on the FAME results obtained with BCP1 cells grown on the three C sources. A one-way ANOVA was performed to test the null hypothesis that there were no significant differences in the mean of standard deviation of the fatty acid composition obtained on the three C sources, followed by Tukey’s *post hoc* test. The results obtained were verified by performing a two-sample *t*-test within pairs of strains. Results reflect three experimental replicates for each growth condition.

### Chemicals and Reagents

The naphthenic acids and the commercially available *cis-* and *trans-* FAME used as reference for GC were purchased from Sigma-Aldrich (St. Louis, MO, United States), except for: 6 *cis*-hexadecenoic acid methyl ester, commercially available from Lipidox (Lidingö; Sweden); methyl 10-methylhexadecanoate, methyl 10-methylheptadecanoate and methyl 10-octadecanoate commercially available from Larodan (Solna, Sweden). Solvents used for FAME analysis such as chloroform, methanol, diethyl ether and *n*-hexane (HPLC grade) were purchased from Baker (New Jersey, United States) and used without further purification.

## Results

### Growth of *R. aetherivorans* BCP1 on NAs

**FIGURE 1:**
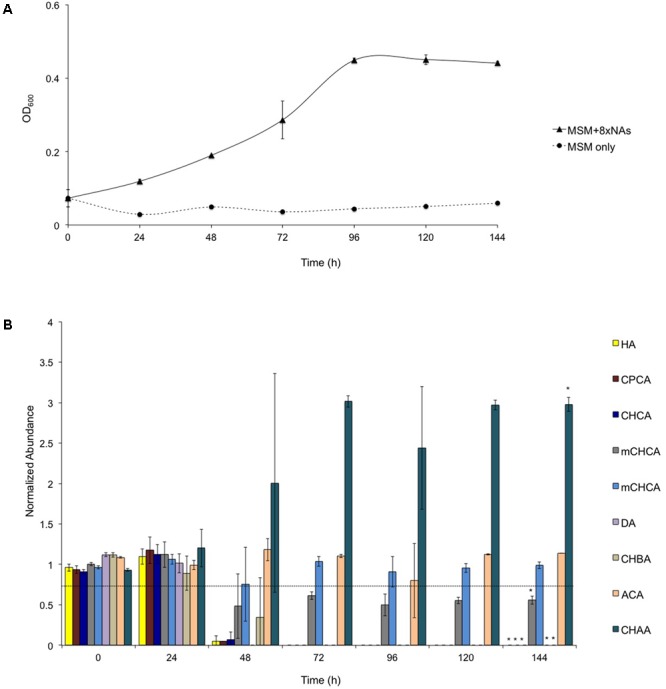
Growth of *Rhodococcus aetherivorans* BCP1 during the utilization of the 8XNA mixture **(A)** and residual concentration of individual components of the 8XNA mixture over time **(B)**. GC-FID was used to determine normalized abundance values relative to an internal standard (*n* = 3). Error bars represent standard error of the mean. A normalized abundance value of ‘1.0’ represents starting NA levels. The final OD_600_ value reached with 8XNAs is significantly different from the final value reached by the control culture, i.e., the BCP1 culture w/o the addition of 8XNAs upon two-factor ANOVA with replication test (*P* < 0.01). Asterisks above the 144 h data denote that the concentration of each NA detected at 144 h is significantly different from that revealed at the beginning of the experiment (0 h) based upon two-factor ANOVA with replication test (*P* < 0.01).

To investigate the ability of *R. aetherivorans* strain BCP1 to grow on NAs as sole carbon and energy sources, cultures of BCP1 cells were initially inoculated in MSM in the presence of an equimolar mixture of eight NAs (notated as 8XNAs, Supplementary Table S1) added at an initial working concentration of 500 mg L^-1^. The residual amount of each NA in the medium and the BCP1 cell growth was periodically assessed over 144 h via GC-FID and OD_600_ measurement, respectively. BCP1 could utilize for growth most of the NAs included in the 8XNA mixture, and reached an OD_600_ of 0.45 in 96 h (**Figure 1A**). In particular, between 48 and 72 h of growth, the aliphatic NAs DA and HA were removed from the medium below detectable limits along with the alicyclic NAs CHCA, CPCA, and CHBA (**Figure 1B**). On the other hand, only one of the two mCHCA isomers was approximately 50% consumed after 48 h without being further degraded; conversely, BCP1 cells were not able to degrade both CHAA and the most recalcitrant ACA (**Figure 1B**). Among these, the level of CHAA increased after 48 h of growth on 8XNAs, as CHAA is a likely metabolic intermediate of CHBA after the removal of an acetate moiety from the butyric side chain.

**FIGURE 2:**
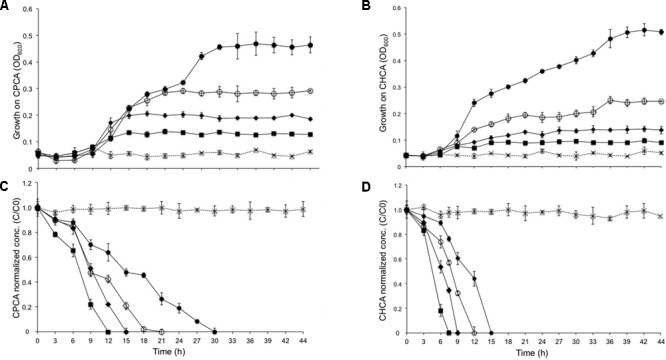
Growth of *R. aetherivorans* BCP1 during the utilization of CPCA **(A)** or CHCA **(B)** at different concentrations (filled squares for 50 mg L^-1^, filled diamonds for 100 mg L^-1^, empty round circles for 200 mg L^-1^, filled round dots for 500 mg L^-1^) and corresponding degradation of the two NAs over time (**C** for CPCA and **D** for CHCA). In the degradation graphs, a normalized abundance value of ‘1.0’ represents starting NA levels. Error bars represent standard error of the mean (*n* = 3). The growth and degradation curves for the sterilized controls are shown as cross marks. The final OD_600_ values (44 h) reached with all the NA concentrations tested (except for CHCA 50 mg L^-1^) are significantly different from the final OD_600_ value reached by the control culture, i.e., the BCP1 culture w/o the addition of any NA based upon two-factor ANOVA with replication test (*P* < 0.01).

Based on these results, we selected two alicyclic NAs, i.e., CHCA and CPCA, that were depleted by BCP1 below detectable limits from the mixture of 8XNAs, to investigate in detail. Biodegradation was evaluated by spiking each individual NA to BCP1 cultures in MSM at four different concentrations (50, 100, 200, and 500 mg L^-1^). As shown in **Figure 2**, BCP1 cells were able to grow on and degrade both the NAs up to the maximal concentrations tested and providing biomass yields of 0.641 ± 0.002 mg_cdw_/mg_(CHCA consumed)_ and 0.881 ± 0.001 mg_cdw_/mg_(CPCA consumed)._ Increasing the concentrations of either NA positively influenced BCP1 growth (in terms of final OD_600_), the resulting growth rate (calculated in the exponential phase) fell between 0.11 and 0.25 h^-1^ for CPCA and between 0.08 and 0.27 h^-1^ for CHCA (Supplementary Figure S1). Despite both the final biomass yields and the growth rates were similar, it was apparent that CPCA was degraded at a lower rate as compared to CHCA, this variation possibly due to metabolic constraints toward initial CPCA degradation or to the higher toxicity of CPCA as compared to CHCA as also manifested by the significant lag phase of CPCA cultures (**Figure 2**). This phenomenon was investigated further by cell flow cytometry. Viable and dead cell counts were determined in BCP1 cultures that were supplied with CHCA (500 mg L^-1^), CPCA (500 mg L^-1^), or glucose (0.1% w/v) as sole carbon/energy sources and analyzed at three different time points. The results, collected in Supplementary Table S4, show that at the beginning of the growth phase (3 h), approximately 95% of the cells in the control cultures (with glucose) and 40% of the cells in CHCA and CPCA-grown cultures were viable, suggesting a significant damage of the cytoplasmic membrane permeability by the toxic NAs or by possible toxic metabolic intermediates. The percentage of damaged and dead cells decreased drastically with CHCA after 9 h of growth reaching viability values similar to the control culture. On the contrary, after 9 h of growth on CPCA, the viable cells were only 60%, indicating still a significant portion of the cells affected by damage on the cell membrane. At an advanced growth phase (30 h), with no or low amounts of NAs present in the medium, a strong recovery of the cytoplasmic membrane integrity in NA-grown cells was apparent, with more than 80% of cells viable, a percentage approaching that measured in control cultures (Supplementary Table S4).

### Cell Morphology Characteristics of *R. aetherivorans* BCP1 Grown on Glucose and NAs

**FIGURE 3:**
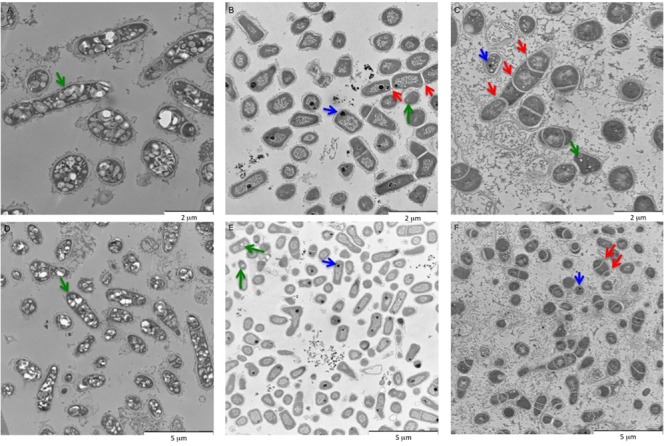
Representative TEM pictures of BCP1 cells grown on glucose **(A,D)**, CHCA (500 mg L^-1^) **(B,E)** or CPCA (500 mg mL^-1^) **(C,F)** till exponential phase. Arrows indicate structures depicted in NA-grown cells as described in detail in the text. Blue arrows = PolyP accumulation bodies; Red arrows = uneven division septa; Green arrow = electron-transparent inclusion bodies.

Transmission electron microscopy (TEM) was performed to analyze the morphology (exponential phase) of BCP1 grown on NAs or glucose. Compared to cells grown on glucose, the prevalent shape for cells growing on NAs corresponded to short rods or cocci (**Figure 3**). In agreement with this, the average size of the cells grown on glucose was 1.70 × 0.98 μm (length × width), while the average size of the cells grown on CHCA and CPCA was 0.98 × 0.69 μm and 1.15 × 0.87 μm, respectively. Further, mainly CPCA- but also CHCA-grown long and short rod cells exhibited irregular shape and an increased number of division septa (**Figures 3B,C,F**). The presence of unresolved division septa has been often associated to uneven or incomplete cell division as a consequence of an altered bacterial cell cycle (Veeranagouda et al., 2009). CPCA grown cells were also surrounded and connected through a diffuse matrix which could be either glycocalyx or exopolymeric substances (EPS) (**Figures 3C,F**). EPS-like material was also present in images of glucose and CHCA-grown cells although it was mainly localized around cells (less dispersed) and connected to the outer layer of the cell membranes.

**FIGURE 4:**
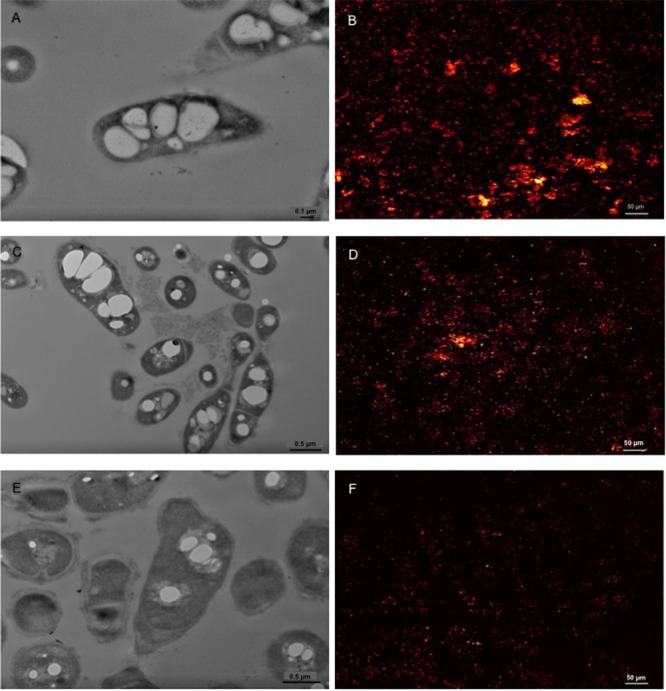
Representative TEM pictures of BCP1 cells grown on glucose (1% w/v; **A**), CHCA (500 mg L^-1^; **C**) or CPCA (500 mg L^-1^; **E**) under nitrogen depletion conditions and the corresponding CLSM images displaying cells treated with the lipophilic dye Nile Blue A (glucose, **B**; CHCA, **D**; CPCA, **F**). The picture of BCP1 cells grown on LB is shown in Supplementary Figure S3.

The morphological analyses indicated the presence of both electron-transparent and electron-dense inclusion bodies in the cells. Spherical, electron-dense inclusion bodies (max two per cell) were observed throughout all growth conditions, although inclusion bodies of cells growing on CHCA were larger than those growing on either CPCA or glucose (**Figures 3B,C,E,F** and Supplementary Figure S2). Growth on glucose was featured by numerous electron-transparent inclusion bodies of different shapes and sizes (**Figures 3A,D**). In CPCA- and CHCA-grown cells some small electron-transparent inclusion bodies were also present, although they were less evident and not visible in all cells (**Figure 3**). While electron-transparent inclusions generally correspond to intracellular accumulation of lipids and lipophilic compounds, the electron-dense inclusion bodies may represent polyphosphate (PolyP) granules. Under conditions of N-depletion (nitrogen-depleted MSM supplied with glucose, CHCA or CPCA as only carbon source), the electron-transparent inclusion bodies were more evident in CHCA- and CPCA-grown cells (**Figures 4C,E**). Fluorescence microscopy using Nile Blue A staining confirmed the presence of intracellular inclusion bodies and the bright orange fluorescence detected in cells grown on glucose and NAs indicated the accumulation of storage lipophilic compounds (**Figures 4**) (Ostle and Holt, 1982; Spiekermann et al., 1999; Oshiki et al., 2011; Weissgram et al., 2015). In particular, fluorescent cell aggregates were visible under glucose and CHCA growth conditions. Red signals were ascribed to cell membranes in the background, as confirmed by the imaging of BCP1 cells grown in LB (Supplementary Figure S3).

### Fatty Acid Composition of *R. aetherivorans* BCP1 Grown on Glucose and NAs

Based on changes in BCP1 cell morphology during growth on NAs, we were interested to gather information on lipid species, which have structural and functional roles. The effects of CHCA and/or CPCA were compared to glucose examining the total fatty acid composition of BCP1 cells grown until exponential phase. In this work, we focused our analysis in order to address the fatty acid biosynthesis under NAs- or glucose-growth conditions, and we did not deepen the quantification of the lipid species, such as the balance between phospholipid and triglyceride species, which can indicate the propensity to membrane formation or lipid accumulation, respectively. However, the crude lipid extract was examined by thin layer chromatography (TLC) to identify the lipid species by elution and comparison with the appropriate reference compounds. Using this analysis we found that triglycerides are present in cells grown on NAs and not in cells grown on glucose. **Table 1** summarizes the results of total fatty acid composition in BCP1 cells under the three growth conditions. Supplementary Figures S4, S5 display representative GC and GC-MS analysis results. GC-MS analyses confirmed the different types of fatty acids in the total extracts, with saturated and monounsaturated fatty acids, C16:0 and C18:1-9c, as prevalent components in the total fatty acid composition of BCP1 cells (**Table 1**). It is worth noting that no formation of *trans-* fatty acids was found, which excludes that such pathway known for bacteria resistance is activated in BCP1 under the growth conditions tested in this work (Chatgilialoglu et al., 2014). Changes of fatty acid patterns among cells grown on glucose and on NAs concerned specific fatty acids: saturated methyl-branched fatty acids (SMBFA), such as 10-methylhexadecanoic (10Me-16:0) and methyloctadecanoic acid (10Me-18:0), were higher in cells grown on NAs as compared to glucose, whereas the opposite trend was found for the saturated fatty acids (SFA) with 16 carbon atoms (C16:0, palmitic acid) and with 18 carbon atoms (C18:0, stearic acid). In the polyunsaturated fatty acid (PUFA) family, the only fatty acid detected was linoleic acid (omega-6, 9cis,12cis-C18:2) that increased in NAs-grown cells as compared to glucose, although the difference resulted significant (*n* = 3, *p* < 0.05) only with CPCA (Supplementary Table S5). It is worth noting that CPCA-grown cells compared to glucose and CHCA showed the increase of the relative abundance of fatty acids with C numbers <16 (**Table 1**). Further, a slightly higher amount of odd-numbered fatty acids was produced by BCP1 cells grown on CPCA, while higher amounts of even-numbered fatty acids were produced by those grown on CHCA, as compared to the other NA and to glucose.

**Table 1:** Composition (% total fatty acids) of *Rhodococcus aetherivorans* BCP1 cell fatty acids grown on glucose, CHCA, or CPCA as the sole carbon and energy source.

Fatty acid	Carbon source					
	Glucose		CHCA		CPCA	
	Mean^a^	ds^b^	Mean^a^	ds^b^	Mean^a^	ds^b^
14:0	1.93	0.68	1.70	0.66	4.77	2.39
15:0	0.65	0.29	0.13	0.02	1.58	0.40
16:0	38.06	1.08	32.48	1.11	27.81	0.29
16:1-6c	1.25	0.47	1.39	1.09	0.90	0.49
16:1-9c	5.64	2.38	4.00	2.07	5.13	0.56
10Me-16:0	3.07	1.52	4.89	1.96	5.50	3.77
17:0	<0.1	–	<0.1	–	<0.1	–
10Me-17:0	3.18	1.89	2.10	0.92	3.45	1.39
18:0	5.93	1.63	2.85	0.75	4.39	0.33
18:1-9t	0.12	0.01	0.21	0.07	0.17	0.04
18:1-9c	24.96	1.85	30.24	3.43	22.54	0.97
18:1-11c	2.45	0.53	2.64	0.93	3.42	1.66
10Me-18:0	9.48	1.03	11.72	2.11	12.60	2.96
18:2-9c,12c	2.69	1.17	5.08	1.58	7.25	1.24
SFA^c^	46.57	3.40	37.17	2.09	38.55	2.51
MUFA	34.41	0.69	38.36	4.19	32.17	1.39
SMBFA	15.73	3.88	18.72	4.16	21.55	0.86
PUFA	2.69	1.17	5.08	1.58	7.25	1.24
C < 16^d^	2.58	0.73	1.82	0.65	6.35	2.42
C > 16	96.82	4.66	97.62	5.62	93.18	5.56
Odd	3.83	1.91	2.23	0.92	5.03	1.44
Even	95.57	4.31	97.21	5.58	94.50	5.89

*^*a*^The mean value from three independent experiments.*

*^*b*^The standard deviation.*

*^*c*^SFA, saturated fatty acids; SMBFA, saturated methylated branched fatty acid; MUFA, mono-unsaturated fatty acids; PUFA, polyunsaturated fatty acids.*

*^*d*^C < 16 = *Σ*fatty acid with carbon n° < 16, C > 16 = Σfatty acid with carbon n° > 16.*

### *In Silico* Analysis of the Putative Genes Involved in NA Degradation and Semi-Quantitative RT-PCR Experiments Targeting These Genes

*Rhodococcus aetherivorans* BCP1 genome analysis was performed to detect the possible genes involved in NA degradation. Using Pathpred software and *in silico* comparative analyses, we identified genes encoding cyclohexanone monooxygenases and genes possibly encoding enzymes involved in oxidative degradation processes (Supplementary Table S3). The expression of these genes was investigated in cells growing on NAs or succinate through semi-quantitative RT-PCR. Additional RT-PCR targets were genes encoding monooxygenases with relaxed substrate range such as *alkB, smo*, and *prm* genes which are known to be induced during BCP1 growth on aliphatic alkanes (Cappelletti et al., 2011, 2015).

**FIGURE 5:**
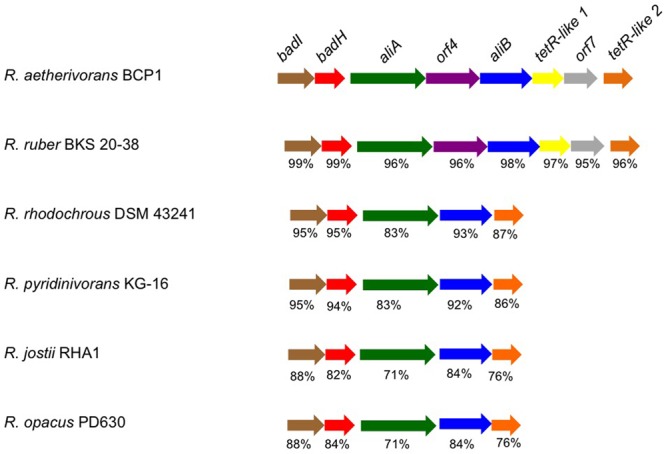
The organization of the *chcpca* gene cluster in *R. aetherivorans* BCP1 and affiliated *Rhodococcus* strains. The % of aa identity is shown below each ORF that shares the same color with the most similar ORF in BCP1.

Among all the BCP1 genes tested in this work, only one gene was found to be induced during growth on CHCA and CPCA as compared to glucose, and it was predicted to code for a 2-hydroxycyclohexanecarboxyl-CoA dehydrogenase (KDE15144) (Supplementary Table S3). This gene (*ORF2* in **Table 2** and *badH* in **Figure 5**) was located in a chromosomal region highly conserved among *Rhodococcus* spp. (**Figure 5**). The predicted products of *ORF2* and of its flanking genes, which were also revealed to be induced by NAs in RT-PCR (KDE15139, KDE15143, KDE15145 genes in Supplementary Table S3), showed amino acid sequences and conserved domains similar to those suggested to be involved in aerobic NA oxidation pathways in other bacteria (Quagraine et al., 2005; Whitby, 2010; Wang et al., 2015). Further, these genes showed similarities (27–53%) with the products of the *bad/ali* genes described in *R. palustris* and previously described to be involved in the metabolism of benzoate and CHCA (**Table 2**) (Pelletier and Harwood, 2000). This gene cluster, which includes *ORF2* and additional genes homologous to *bad/ali* (**Figure 5**), was named as the *chcpca* cluster (**Table 2**). Further quantitative RT-PCR analyses focused on the study of the *chcpca* gene cluster expression.

**Table 2:** ORFs and ORF products included in the *chcpca* gene cluster.

ORFs in *chcpca* cluster	Homologs in β -oxidation	Possible function in NAs degradation	GenBank annotation	GenBank ID
*ORF1*	*badI*	2-Ketocyclohexane 1-carboxyl-CoA hydrolase	Naphthoate sinthase	KDE15145
*ORF2*	*badH*	2-Hydroxycyclohexane-1-carboxyl-CoA dehydrogenase	2-Hydroxycyclohexane-CoA dehydrogenase	KDE15144
*ORF3*	*aliA*	Cyclohexane carboxylate Co-A ligase	Long-chain-fatty-acid-CoA ligase	KDE15143
*ORF4*	–	Glutaryl-CoA dehydrogenase	Glutaryl-CoA dehydrogenase	KDE15142
*ORF5*	*aliB*	Cyclohexanecarboxyl-CoA dehydrogenase	Butyril-CoA dehydrogenase/Long-chain-acyl CoA-dehydrogenase	KDE15141
*tetR-like 1*	–	TetR-like regulator	Transcriptional regulator (TetR Family)	KDE15140
*ORF7*	–	Cyclohex-1-ene 1-carboxyl-CoA hydratase	Enoyl-CoA hydratase	KDE15139
*tetR-like 2*	–	TetR-like regulator	Transcriptional regulator (TetR Family)	KDE15138

### RT-qPCR Analysis of the *chcpca* Gene Cluster

**FIGURE 6:**
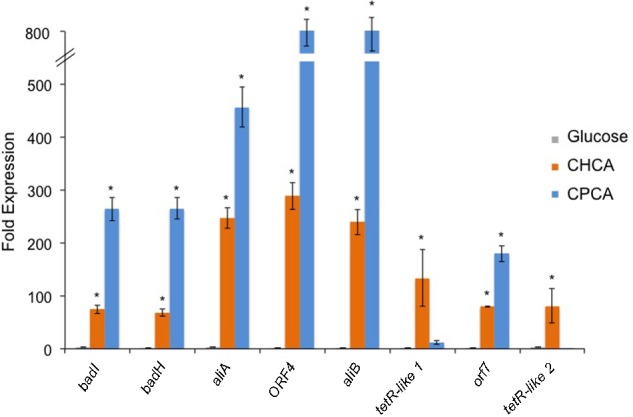
Relative abundance of the transcripts of the eight ORFs from the *chcpca* cluster (shown in **Figure 5**) in *R. aetherivorans* BCP1 cells grown on glucose, CHCA, or CPCA as determined by qPCR. The values obtained for the cultures grown on succinate were used as references. Asterisks denote that the expression values are statistically different from those obtained with succinate based upon one-factor ANOVA with replication test (*P* < 0.01). Data is presented as the mean ± SD of three replicates.

Quantitative reverse transcription-PCR (RT-qPCR) experiments were performed to define the differential transcription of the genes of the *chcpca* cluster during growth on NAs, glucose, or succinate. Succinate was evaluated as an alternative carbon source to glucose as the latter often acts as a catabolite repressor in biodegradative pathways repressing the transcription of catabolic gene operons (Liang et al., 2016). Each gene of the cluster (both catabolic and regulatory) was targeted for quantification. As compared to expression levels during growth on succinate, the levels of ORF1 to ORF5 and Orf7 transcripts were approximately 70 to 290- and 180 to 800-fold higher on CHCA and CPCA, respectively (**Figure 6**). On the other hand, the level of transcripts of the two transcriptional regulators included in *chcpca* cluster, i.e., tetR-like 1 and tetR-like 2 varied between the two different NAs tested; in particular, the two tetR-like genes were transcriptionally induced on CHCA while their levels were similar on CPCA, glucose and succinate (**Figure 6**).

**FIGURE 7:**
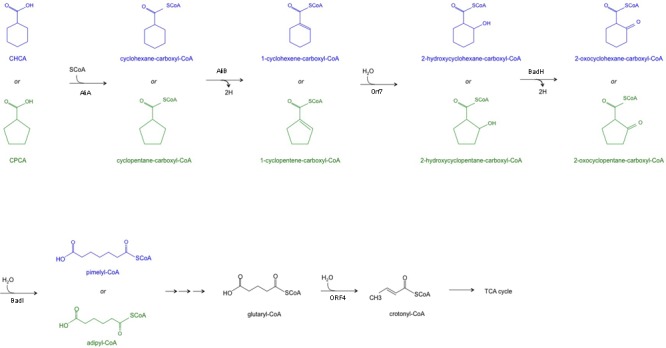
The proposed metabolic pathway of CHCA (blue) and CPCA (green) catalyzed by the enzymes encoded by the *chcpca* gene cluster. A triple arrow indicate that more than one reaction is supposed to occur. TCA = tricarboxylic acid cycle.

These results demonstrate the transcriptional induction of genes included in the *chcpca* cluster during the aerobic growth of BCP1 cells on the two NAs tested, while the two tetR-like genes were apparently involved in only CHCA degradation. On the basis of the similarity of *chcpca* cluster genes with the genes described to be involved in oxidative processes in other microorganisms and with those belonging to the *bad/ali* operon described in *R. palustris*, the *chcpca* cluster genes are proposed to be involved in the β-oxidation pathway included in the biodegradation pathway of CHCA and CPCA in *R. aetherivorans* BCP1, as reported in **Figure 7**.

## Discussion

This study is the first report on the capacity of *R. aetherivorans* BCP1 to degrade aliphatic and alicyclic NAs within a mixture of eight model compounds (8XNAs) supplied at a concentration similar to that reported in Alberta oil sands (Demeter et al., 2014). Interestingly, our data show that *R. aetherivorans* BCP1 cells present a NA degradation profile similar to that seen in microbial communities isolated from oil sands (OSPW) (Demeter et al., 2014), and this suggests the extraordinary degradation capacity of BCP1 strain also toward hydrocarbons different from those on which the strain was originally isolated, i.e., aliphatic alkanes and chlorinated alkanes (Cappelletti et al., 2011, 2015). Further, the biodegradation performance (**Figure 2** and Supplementary Figure S1) was also higher in BCP1 cultures as compared to *Rhodococcus* sp. Y3 and G3 strains, which were isolated from OSPW by Demeter et al. (2014, 2015). In particular, BCP1 degraded the aliphatic compounds DA and HA and the alicyclic compounds CHCA, CPCA and CHBA below detectable limits within 3 days instead of 14 days as reported for the community of organisms and isolates previously cultured from OSPW (Demeter et al., 2015). On the other hand, the BCP1 inability to degrade ACA is correlated to the recalcitrance of the tricyclic adamantane structure to biodegradation which has been previously observed in oil sands tailings-derived microbial communities even in the presence of additives such as peptone, yeast extract, glucose, and molasses (Demeter et al., 2015). Similarly to *Rhodococcus* sp. Y3 and G3 strain, not only CHAA was not degraded by BCP1 but it was also accumulated in the growth medium, suggesting the production of CHAA as a metabolic intermediate during the BCP1 growth on the 8XNA mixture as seen in previous studies on CHBA degradation (Quesnel et al., 2011). The rise of CHAA in the BCP1 culture broth likely derives from the loss of an acetate moiety from the carboxylic acid functional group of CHBA. The ability to degrade CHCA and CHBA, but not CHAA also indicates that biodegradation in BCP1 occurs via β-oxidation alone, and not via a combination of α- and β-oxidation. Indeed, CHAA includes an even-length carboxylic acid as the functional group, which would require α-oxidation for degradation, as β-oxidation is blocked by the tertiary carbon on the ring (Del Rio et al., 2006; Han et al., 2008; Quesnel et al., 2011; Kannel and Gan, 2012).

The growth curve of BCP1 on CHCA and CPCA applied at concentrations up to 500 mg L^-1^ showed an initial lag phase that was absent in the growth curve on glucose (Supplementary Figure S6). In this respect, the longer lag phase observed with CPCA as compared to CHCA is possibly related to a greater toxicity exerted by the first NA. The cell membrane permeability measurements by citofluorimetry and the increased amount of EPS production in CPCA-grown cells as compared to CHCA support this theory (**Figure 3** and Supplementary Table S4). Presumably, the higher toxicity and longer lag phase affected the degradation rate of CPCA as compared to CHCA, while the growth rate during the exponential phase and the final biomass yields were similar in both culture conditions suggesting the presence of similar degradation mechanisms along with a full recovery of the cell membrane integrity in cells grown on both NAs. The production of EPS was proposed to be a key mechanism to the assimilation of alkane substrates at low temperature by *Rhodococcus* sp. strain Q15 and it was described to have an emulsifying effect on alkane cell assimilation and oxidation (Whyte et al., 1999; Bredholt et al., 2002). Further changes in the morphology of BCP1 cells grown on NAs included a slight reduction in cell size and irregular shapes. Modifications in the cell size were previously correlated with stress response in *R. opacus* 1CP and *Rhodococcus* sp. strain DN22 (Priestley et al., 2006; Solyanikova et al., 2017). Conversely, an increase of the cell size was reported with *R. erythropolis* strain IBBPo1 grown on aromatics (Stancu, 2015) in parallel with the accumulation of intracellular electron-transparent inclusion bodies, which are typically hydrophobic compounds with a storage function (Alvarez et al., 1996). Similar electron-transparent inclusions were visible in BCP1 cells grown on MSM with glucose, while CHCA-grown BPC1 cells exhibited prominent electron-dense inclusion bodies, one or two granules per cell near the DNA containing region (**Figure 3** and Supplementary Figure S2), which were smaller and less evident in CPCA-grown cells. These formations are related to polyphosphate (PolyP) granules having a role in increasing bacterial cell resistance to unfavorable environmental conditions (Chávez et al., 2004). In *R. erythropolis* N9T-4, polyphosphate kinase genes were involved in PolyP accumulation occurring only under oligotrophic growth conditions, and resulted in the formation of oligobodies (Yoshida et al., 2016). Oligobodies play various cellular functions in eukaryotic cells such as storage of phosphorus, pH homeostasis, osmoregulation, and stress response (Yoshida et al., 2016). Notably, the oligobodies previously described in N9T-4 cells were significantly similar to those seen in CHCA-grown BCP1 cells (Supplementary Figure S2). Yoshida et al. (2016) demonstrated that PolyP was mainly synthesized by the polyphosphate kinase Ppk1, although there was an additional Ppk2 that did not show sequence similarity to Ppk1. The BCP1 genome possessed a *ppk1* gene encoding a polyphosphate kinase with 77% amino acid identity with the 5′ portion of the *ppk1* gene product of N9T-4. No homolog was present in BCP1 for the N9T-4 *ppk2* gene product (Supplementary Table S6).

Under N-limiting growth conditions the PolyP accumulation bodies had a reduced size in CHCA-growing cells; conversely, both CHCA and CPCA-cells exhibited prominent intracellular electron-transparent accumulations similar to those observed in glucose-grown BCP1 cells (**Figure 4**) and to those observed in other *Rhodococcus* strains grown under N-depletion conditions with different carbon sources (Alvarez et al., 1996). The formation of these inclusion bodies in *R. opacus* PD630 was associated with a cell response to hydrocarbon growth (Alvarez et al., 1996). In BCP1 cells, Nile Blue A dye staining indicated an intracellular accumulation of storage lipids following the cell growth on NAs under N-limiting conditions. *Rhodococcus* BCP1 is taxonomically related to *R. ruber* NCIMB40126 and *R. aetherivorans* IAR1 (Orro et al., 2015), previously shown to prevalently produce PHAs growing on carbohydrates and/or toluene (Haywood et al., 1991; Alvarez et al., 2000; Hori et al., 2009). Both NCIMB40126 and IAR1strains accumulated one type of PHA, the poly-[3-hydroxybutyrate-co-3-hydroxyvalerate] (PHBV) constituted of 3-hydroxyvalerate and 3-hydroxybutyrate monomers. In our case, the bright orange fluorescence observed through microscopy with NAs- and glucose-grown BCP1 was interpreted as evidence for intracellular PHA accumulation, as previously proposed by Ostle and Holt (1982) and Berlanga et al. (2006).

Changes in the BCP1 cellular features during growth on NAs were also apparent from variation of the lipid types and total cellular fatty acid composition. Although a quantification of the lipid species in cells was not performed, the TLC analysis gave qualitative information on triglyceride accumulation in the NAs-grown cells, thus confirming that the reported morphological differences can be combined with such lipid changes. The fatty acid composition could be instead precisely addressed in the total lipid extracts (**Table 1** and Supplementary Figures S4, S5). BCP1 cells grown on NAs as compared to glucose decreased the SFA composition of C16:0 and C18:0 in parallel with increase of the corresponding SMBFAs and the only PUFA detected in the study, 9cis,12cis-C18:2. These changes might reflect the different metabolism of the two short-chain carboxylated cycloalkanes as compared to glucose. On the other hand, 9cis,12cis-C18:2 usually derives from the activity of the delta-12 desaturase on the mono-unsaturated substrate 9cis-C-18:1 (Ferreri and Chatgilialoglu, 2012). In our experiments, we found that the PUFA increase corresponded to an increase of the MUFA content in CHCA-grown cells whereas with CPCA such effect was not present. The bacterial production of this PUFA might be an adaptive response mechanism to the carbon source exchanging the roles of saturated and methyl-branched fatty acids that certainly represent an interesting interplay of biophysical and biochemical effects (Heipieper and de Bont, 1994; Cortes and de Carvalho, 2015). The synthesis of PUFA is very unusual in mesophilic bacteria, while most of the bacteria producing PUFAs are present in marine habitats and cold, deep-sea sediments, fish, and water (Hamamoto et al., 1995). While changes in the saturated fatty acid fraction were related to modification of the membrane fluidity, the increase of methyl-branched fatty acids and PUFA were proposed to participate in the adaptation of *Rhodococcus* spp. to lipophilic compounds and to salt stress (de Carvalho et al., 2014; Cortes and de Carvalho, 2015). Further, *R. erythropolis* DCL14 cells grown on different *n*-alkanes contained a different percentage of each type of fatty acid according to the chain length of the *n*-alkane, the phase of growth and on the medium composition (Cortes and de Carvalho, 2015).

Previous respiration measurements have shown the presence of β-oxidation products as metabolic intermediates of CHCA degradation in *P. putida* and *Acinetobacter* sp. and the inducible nature of these enzymes (Blakley, 1978). Almost nothing is known about the CPCA metabolic intermediates, while a study on the use of cyclopropanecarboxylate (CPrCA) by *R. rhodochrous* indicated that CPrCA is initially converted into its CoA thioester which undergoes the ring-opening reaction in order to be further degraded through the β-oxidation pathway (Toraya et al., 2004). In the present study, the *chcpca* cluster, coding for enzymes involved in β-oxidation reactions, is transcriptionally induced in BCP1 cells utilizing CPCA and CHCA as the only carbon and energy source. Therefore the gene products of the *chcpca* cluster are proposed to be involved in the β-oxidation of CHCA and CPCA as reported in **Figure 7**. An alternative metabolism of CHCA was described in *Corynebacterium cyclohexanicum* and *Arthrobacter* spp. and it was proposed to undergo the aromatization pathway involving the *pobA* gene product for the oxidation of *p*-hydroxybenzoate into protocatechuate acid. RT-PCR analyses showed that the only *pobA* gene detected in BCP1 genome was not transcriptionally induced during the growth on CHCA and CPCA (Supplementary Table S3), excluding the possible involvement of the aromatization pathway in their degradation by BCP1 (Blakley, 1974, 1978).

## Conclusion

This work demonstrates the wide degradation abilities of *R. aetherivorans* BCP1 toward toxic NAs and the involvement of a gene cluster, named *chcpca*, encoding β-oxidation enzymes during NA degradation. These metabolic features linked to accumulation of intracellular inclusion bodies and production of PUFA highlight the possibility of using BCP1 strain in a value-added bioconversion of pollutant NAs.

## Author Contributions

AP carried out the experiments. MC wrote the manuscript and along with AP conceived and planned the experiments. AnS and CF performed the lipidomic analyses and gave support to the interpretation of lipidomic results. MD helped in designing the 8XNAs degradation assay and in editing the final version of the manuscript. EP helped in CLSM analyses. SC and MP carried out the TEM analysis and contributed to microscopy images’ interpretation. GM carried out RT-qPCR experiments. SF contributed to the statistical analysis of FAME results. AlS hosted in his lab and supported AP while he was at the University of Münster where he carried out RT-PCR experiments. DZ and RT financially supported the project, provided critical feedback, and helped to shape the manuscript. All authors provided critical feedback and contributed to the final manuscript.

## Conflict of Interest Statement

The authors declare that the research was conducted in the absence of any commercial or financial relationships that could be construed as a potential conflict of interest.
