# Supplementary material for: Aerobic Growth of Rhodococcus aetherivorans BCP1 Using Selected Naphthenic Acids as the Sole Carbon and Energy Sources
Source: Front Microbiol. 2018 Apr 12;9:672. doi: 10.3389/fmicb.2018.00672 (PMC5906575; doi:10.3389/fmicb.2018.00672)
Supplement: Supplementary file 1 [file Data_Sheet_1.pdf]

frontiers

Supplementary Material

Aerobic growth of *Rhodococcus aetherivorans* BCP1 using selected naphthenic acids as the sole carbon and energy sources

Presentato A[† 1,2], Cappelletti M[†*1], Sansone A[3], Ferreri C[3], Piacenza E[2], Demeter M[2], Crognale S[4], Petruccioli M[4], Milazzo G[1], Fedi S[1], Steinbüchel A[5,6], Turner RJ[2§], Zannoni D[1§]

† These authors contributed equally to the work.
§ These authors share senior authorship.

*** Correspondence:**
Dr Martina Cappelletti, Department of Pharmacy and Biotechnology, University of Bologna, Via Irnerio 42, 40126 Bologna, Italy, martina.cappelletti2@unibo.it, marty.cap@gmail.com

Supplementary Tables

Supplementary Table S1 Constituent model NAs contained within the 8χNA mixture at equimolar (1.7 mM) concentrations and water solubility values[a]

Naphthenic Acid	Abbrev.	Structure
Cyclohexane carboxylic acid	CHCA	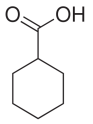
Cyclohexane acetic acid	CHAA	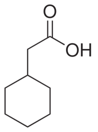
Hexanoic acid	HA	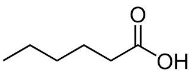
Decanoic acid	DA	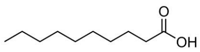
3-Methyl-1-cyclohexane carboxylic acid (*cis* and *trans*)	mCHCA	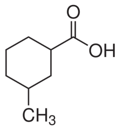
1-Adamantane carboxylic acid	ACA	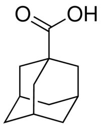
Cyclohexane butyric acid	CHBA	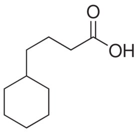
Cyclopentane carboxylic acid	CPCA	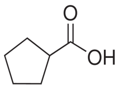

[a] The stock solutions of each NA is prepared as sodium salt naphthenate which is described in the literature to be soluble in water (see Material and Methods).

Supplementary Table S2 RTqPCR Primers used for RT-qPCR experiments of *chcpca* gene cluster and the 16S rRNA gene.

Orfs	**Locus tag**	**Forward Primer**	**Reverse Primer**
orf1	N505_0103275	tctcggcgatcatcctcac	atctcgaacatgccgctct
orf2	N505_0103270	atcaacgtcaactgcgtgtg	aacgggatggccttgatca
orf3	N505_0103265	gatgctctcgtcgttcaagc	gtggccttgtaatgcagctt
orf4	N505_0103260	cttcggcaagctcaacaaca	gatgacggggtactcgagg
orf5	N505_0103255	tacaacgccatcctgatcga	tggtcatgtcggtcaggtac
tetR-like 1	N505_0103250	cgcacagggttccatcagat	ctcctccttgttgccgaagt
orf6	N505_0103245	gcgatgatggagtcctggag	gatgcgggcgaacacgtc
tetR-like 2	N505_0103240	gtgatgaacccggtcgatgt	gtgtagatggccccgaagtg
16S rRNA	N505_0111375	attagtggcgaacgggtgag	cccgaggtcctatccggtat

Supplementary Table S3 List of ORFs (locus tags and gene products) analysed through semi-quantitative RT-PCR and corresponding transcriptional inducti results obtained on CPCA, CHCA and succinate.

Product (GenBank)	Locus tag	Protein ID	Forward Primer	Reverse Primer	CPCA	CHCA	Succinate	Inducer[b]
Cyclohexanone monooxygenase	N505_0100500	KDE14614	gtgacctcgatggtgtggaa	gcacgtcgatctcgtactcg	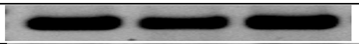			ND
	N505_0100750	KDE14662	gcagccggtacttcatctcc	aagtcgacgtggtcctcgat	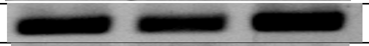			ND
	N505_0105690	KDE14308	gggcgagatcttccactcc	tgcaggttgcggttgtattc	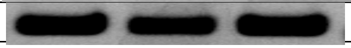			ND
	N505_0118125	KDE12215	ctgccgtacttccagcagtg	cgcagcatcgtccagtagtc	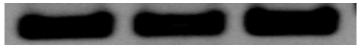			ND
	N505_0118260	KDE12242	tcgaggtcgacgagagtgaa	atgaacggcatgaagacacg	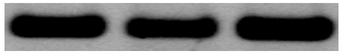			ND
	N505_0119480	KDE11803	gcaagatgctcggggactac	tcgaagaacgggaaggtgat	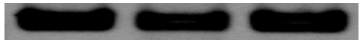			ND
	N505_0122350	KDE10976	ccggccctgtactcgtactc	ggagctggctctcgatcat	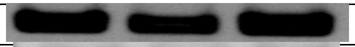			ND
	N505_0122450	KDE10995	cctggttctggaaccgctat	cccttgtccgtgaactcctc	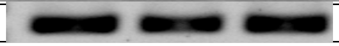			ND
	N505_0122535	KDE11012	gacgtggacagccacgacta	gcggttgaaggtctcgtacc	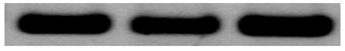			ND
	N505_0125330	KDE11542	ctgcagcagtggaactggac	gatgttggtgaacgggctct	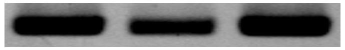			ND
	N505_0125485	KDE11570	ggactggagccgaatctttg	gcatgtcgaggatgtggaag	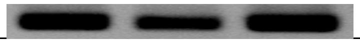			ND
	N505_0127890	KDE10307	gcggcaagatgttccactc	gggacgggtcacgtagttct	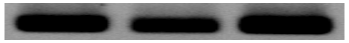			ND
p-Hydroxybenzoate hydroxylase	N505_0123160	KDE11135	agcacgaggtcaacaaggac	gacatccaccacgagaagtg	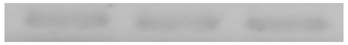			ND
2-Hydroxycyclohexane-CoA dehydrogenase	N505_0118185	KDE12227	cttcgacctgaacggaaagg	ctcggagcacagccacag	ND	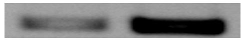		ND
	N505_0103270	KDE15144	atgagcaacatcgcactggt	gaacgggatggccttgat	ND	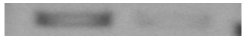		ND
Long-chain-fatty-acid-CoA ligase	N505_0118930	KDE11696	ccgacatgttgaccgaggta	cggtggtagggagttgatcc	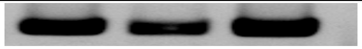			ND
	N505_0103265	KDE15143	cgacaccgaactcaccgata	aacttggccctctcctggtc	ND	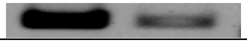		
Enoyl-CoA hydratase	N505_0107140	KDE14076	aactgatgcgtgaggtggtg	gccatgccttccttctggt	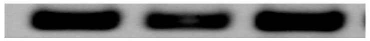			ND
	N505_0103245	KDE15139	gtcaccacacgcgaagacc	gacgaatccttccagcaagc	ND	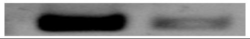		
Naphthoate synthase	N505_0118865	KDE11684	ctgatttcaccgcattcacg	agcgactgcttgagcacctt	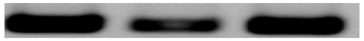			ND
	N505_0103275	KDE15145	gaccgtgttcgaggacatca	gaggccctcgtcggactt	ND	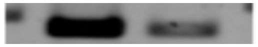		ND
Alkane 1-monooxygenase (AlkB)	N505_0120250	KDE10575	tacatcgagcacaaccgcggc	tgacgatgtggtcggagtt	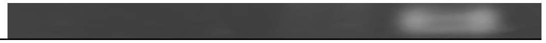			
Propane monooxygenase (PrmA)	N505_0124220	KDE11344	gtacggcaccaaggaccgcc	gagggtcttgccgtcgtcgc	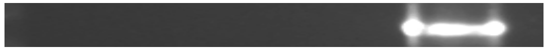			
16S rRNA[a]	N505_0111375		agagtttgatcmtggctcag	tacggytaccttgttacgactt	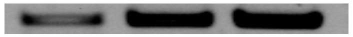			ND

[a] Gene retrotranscribed as a positive control of the RT-PCR experiment
[b] Substrate that has been detected as transcriptional inducer in previous studies; hexane (C_6) for *alkB* gene, propane (C_3) for *prm* gene

Supplementary Table S4 Percentage of damaged or dead cells obtained through cytofluorimetry analysis of BCP1 cell cultures grown on glucose (0.1% w/v) or NAs (500 mg/L)[a]

Growth substrate	Hours of growth			
	0 h	3 h	9 h	30 h
Glucose	5.5 %	5.4 %	5.0 %	9.6 %
CPCA	7.0 %	59.0 %	39.5 %	19.7 %
CHCA	6.6 %	58.5 %	4.4 %	5.8 %
None (MSM only)	6 %	-	-	14.0 %

[a] The % of dead cells was measured as number of [dead cells (red fluorescent)]/ [viable cells (green fluorescent) + dead cells (red fluorescent)] * 100

Supplementary Table S5 ANOVA test performed on fatty acid composition of BCP1 cells grown on glucose, CHCA and CPCA[a]

	GLU/CHCA	CHCA/CPCA	GLU/CPCA
C 14:0	NS[b]	NS	NS
C 15:0	NS	$P < 0.05$	NS
C 16:0	$P < 0.05$	$P < 0.05$	$P < 0.05$
16:1 6 C	NS	NS	NS
16:1 9 C	NS	NS	NS
Me 16:0	NS	NS	NS
Me 17:0	NS	NS	NS
18:0	NS	NS	NS
18:19 t	NS	NS	NS
18:19 C	NS	NS	NS
18:1 11 C	NS	NS	NS
Me 18:0	NS	NS	$P < 0.05$
18:2	$P < 0.05$	NS	NS
SFA	NS	NS	NS
SMBFA	NS	NS	$P < 0.05$
MUFA	NS	NS	NS
PUFA	NS	NS	NS
C<16	NS	NS	NS
C>16	NS	NS	NS
ODD	NS	NS	NS
EVEN	NS	NS	NS

[a] A one-way ANOVA was performed to test the null hypothesis that there were no significant differences in the mean of s.d. of the fatty acid composition obtained with the three C sources, followed by Tukey's post-hoc test. The results obtained were verified by performing a two-sample *t*-test within pairs of strains. Results reflect three experimental replicates for each growth condition.
[b] NS=not statistically significant

Supplementary Table S6 Genes encoding enzymes possibly involved in TAG, PHA, and PolyP accumulation in *R. aetherivorans* BCP1. NCBI annotation is indicated as well as the Accession number.

Gene name	Enzyme name	Accession #
phaC	PHA synthase	KDE13084<br>KDE10636<br>KDE13191<br>KDE11780
phaZ	PHA depolymerase	KDE11778<br>KDE10868
ppk	Polyphosphate kinase	KDE14014
ppx	Exopolyphosphatase	KDE15351<br>KDE13580<br>KDE12358
atf	Wax ester synthase/acyl-CoA:diacylglycerol acyltransferase	KDE15033<br>KDE14033<br>KDE13744<br>KDE13626<br>KDE13627<br>KDE12630<br>KDE13739<br>KDE10687

Supplementary Figures

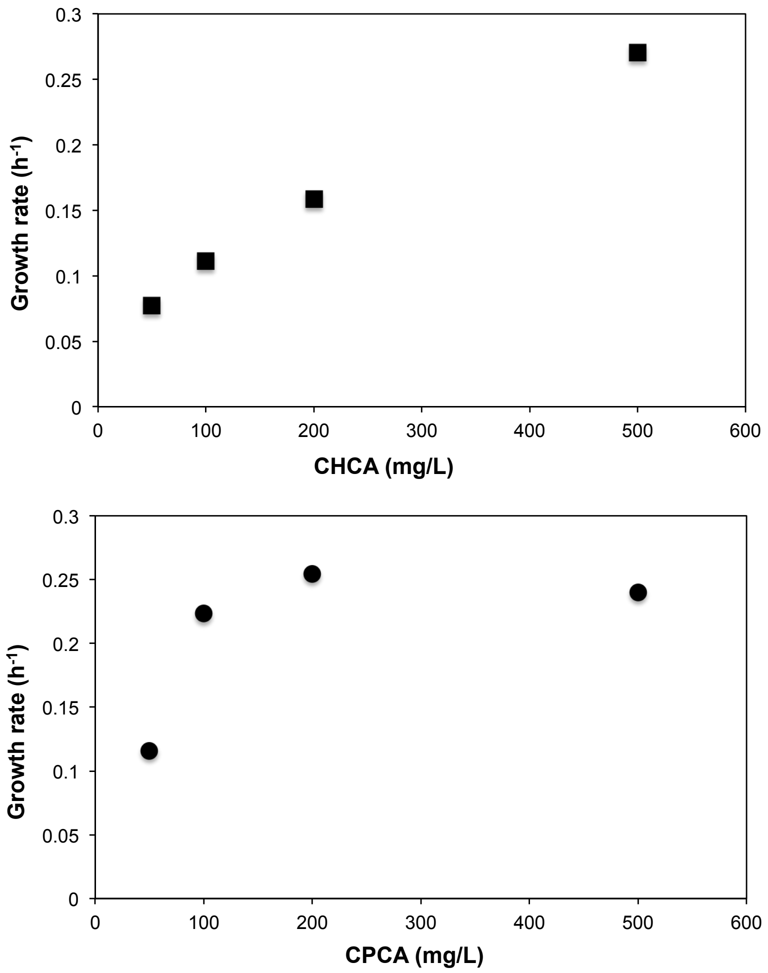

Supplementary Figure S1. Growth rate (h^{-1}) variation according to the different concentrations of CHCA and CPCA supplied to BCP1 cells growing on MSM.

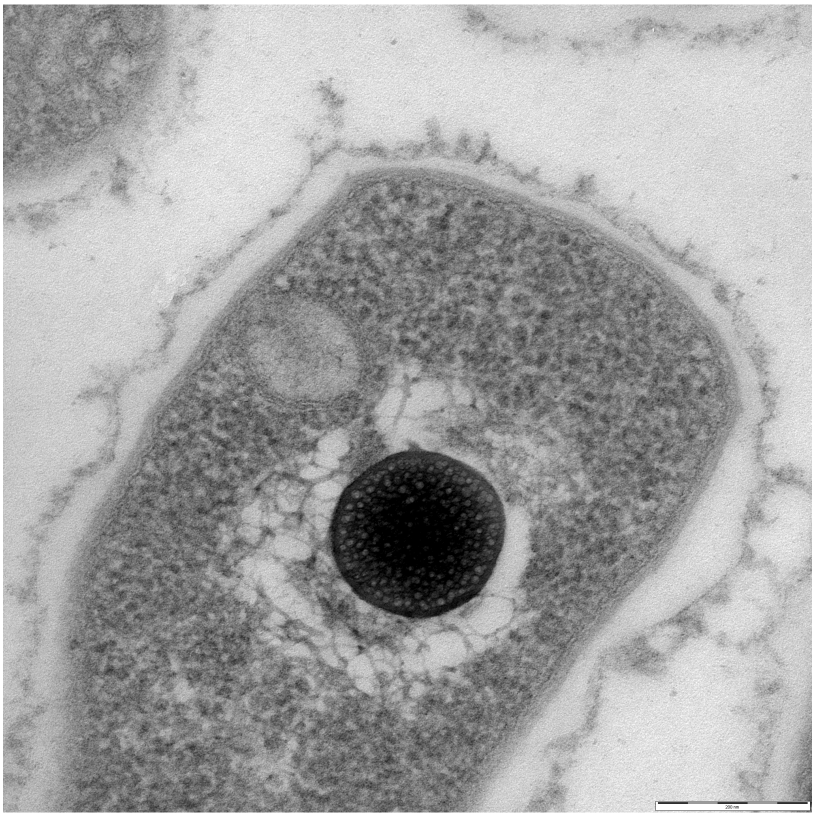

Supplementary Figure S2. TEM image showing an intracellular oligobody in a BCP1 cell grown on CHCA (500 mg/L)

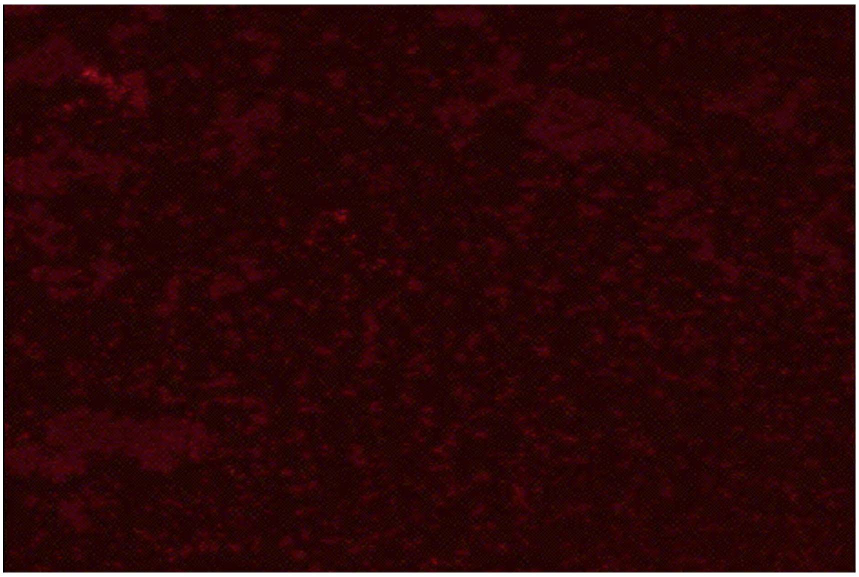

Supplementary Figure S3. CLSM image of BCP1 cells grown in LB until exponential phase that were stained with Nile Blue A.

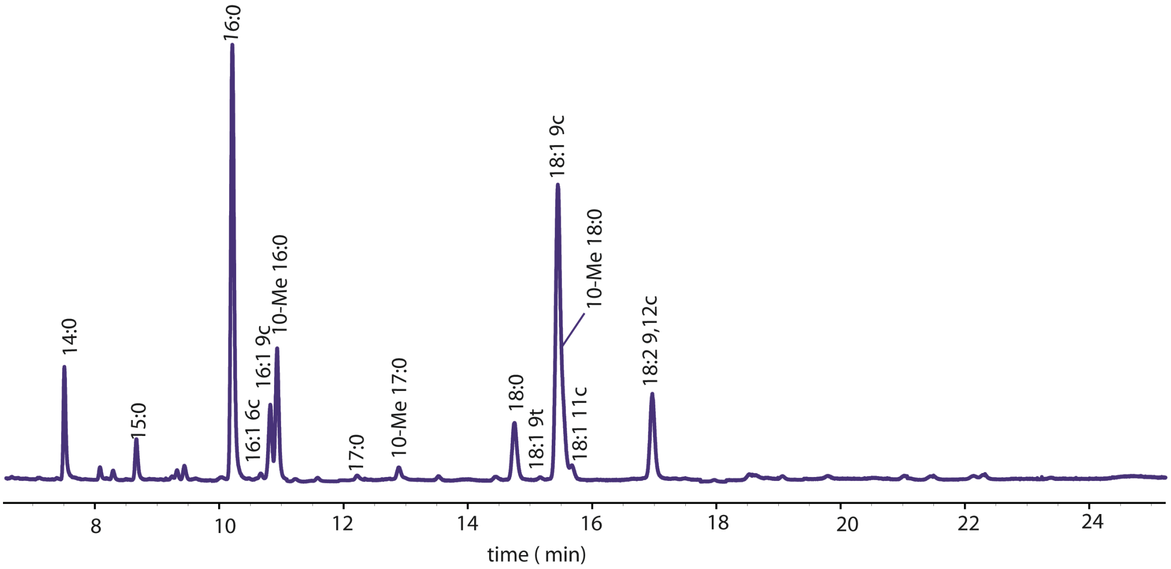

Supplementary Figure S4. Representative GC analysis of FAME obtained from BCP1 grown on NAs. The FAME were identified by comparison with the retention times of reference compounds.

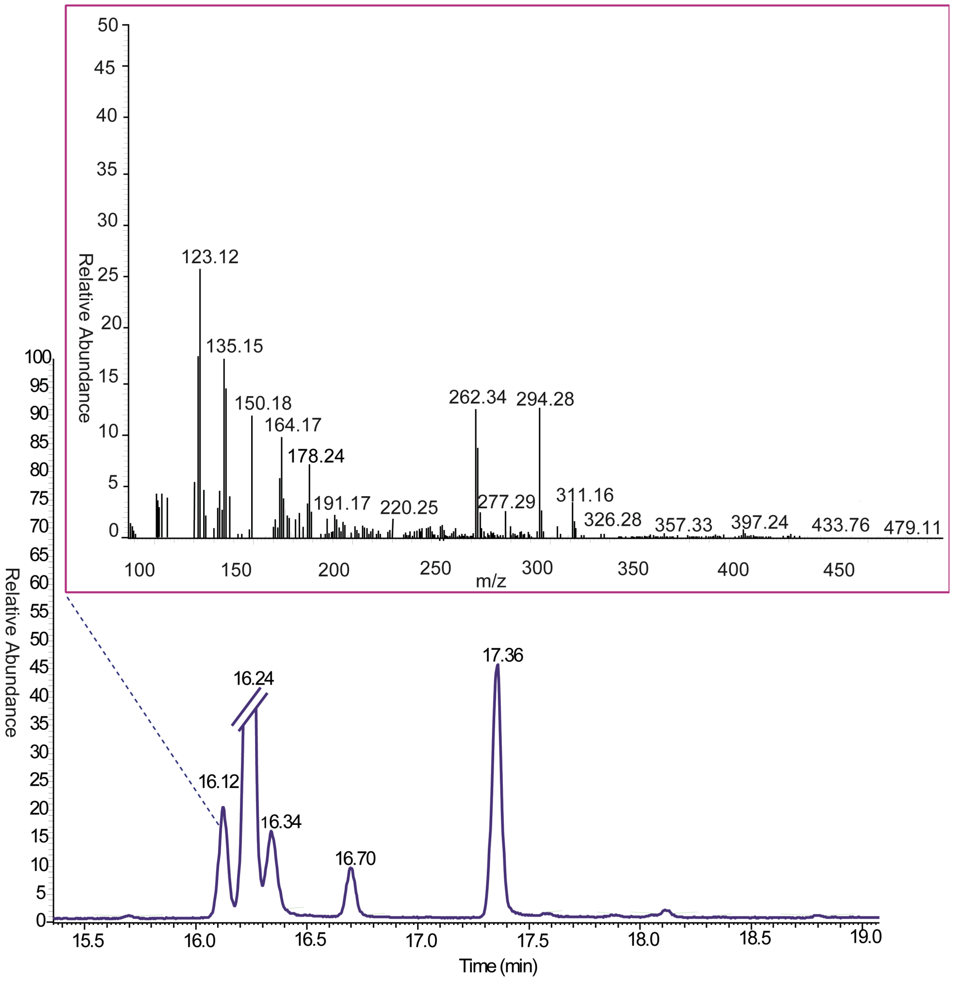

Supplementary Figure S5. Section of GC-MS analysis of FAME obtained from BCP1 grown on NAs. In the box is reported the MS of the peak eluted at 16:12 min confirming the presence of linoleic acid (18:2 9c,12c.) The rest of the reported peaks corresponded to the methyl esters of oleic acid (16.24 min), vaccenic acid (16.34 min), stearic acid (16.70 min) and methyl-10 octadecanoic acid (17.36 min).

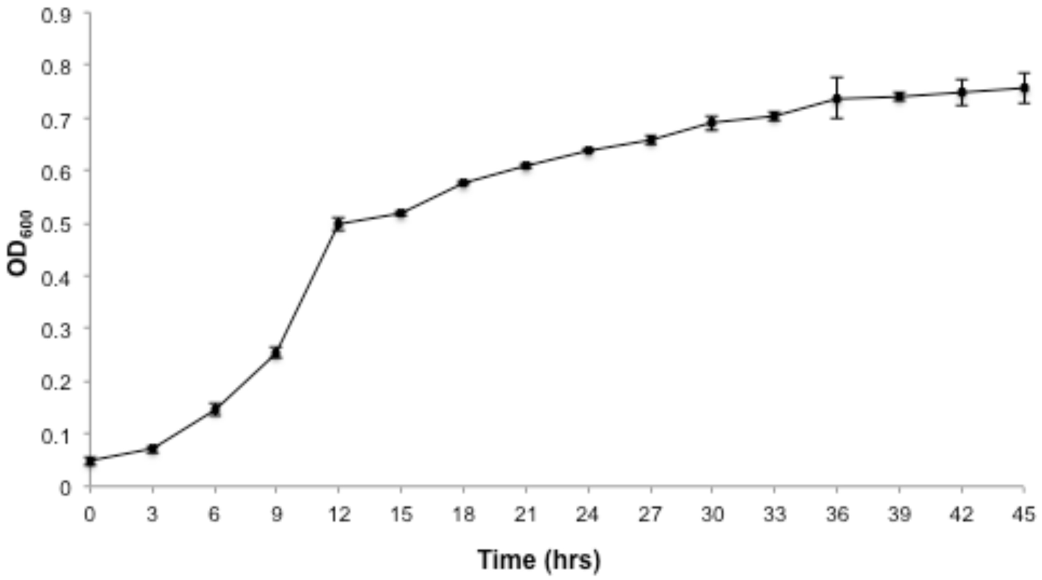

Supplementary Figure S6. Growth curve of BCP1 on MSM supplied with glucose (0.1% w/v)
